# Association study of the vesicular monoamine transporter 1 (VMAT1) gene with schizophrenia in a Japanese population

**DOI:** 10.1186/1744-9081-2-39

**Published:** 2006-11-30

**Authors:** Misty Richards, Yoshimi Iijima, Hitomi Kondo, Tomoko Shizuno, Hiroaki Hori, Kunimasa Arima, Osamu Saitoh, Hiroshi Kunugi

**Affiliations:** 1 Department of Mental Disorder Research, National Institute of Neuroscience, National Center of Neurology and Psychiatry, Tokyo, 187-8502, Japan; 2 Albany Medical College, Albany, NY 12208, USA; 3 Department of Psychiatry, Musashi Hospital, National Center of Neurology and Psychiatry, Tokyo, 187-8502, Japan

## Abstract

**Background:**

Vesicular monoamine transporters (VMATs) mediate accumulation of monoamines such as serotonin, dopamine, adrenaline, and noradrenaline from the cytoplasm into storage organelles. The VMAT1 (alternatively solute carrier family 18: SLC18A1) regulates such biogenic amines in neuroendocrine systems. The VMAT1 gene maps to chromosome 8p21.3, a locus with strong evidence of linkage with schizophrenia. A recent study reported that a non-synonymous single nucleotide polymorphism (SNP) of the gene (Pro4Thr) was associated with schizophrenia.

**Methods:**

We attempted to replicate this finding in a Japanese sample of 354 schizophrenics and 365 controls. In addition, we examined 3 other non-synonymous SNPs (Thr98Ser, Thr136Ile, and Val392Leu). Genotyping was performed by the TaqMan allelic discrimination assay.

**Results:**

There was no significant difference in genotype or allele distribution of the three SNPs of Pro4Thr, Thr136Ile, or Val392Leu between patients and controls. There was, however, a significant difference in genotype and allele distributions for the Thr98Ser polymorphism between the two groups (*P *= 0.01 for genotype and allele). When sexes were examined separately, significant differences were observed in females (P = 0.006 for genotype, P = 0.003 for allele), but not in males. The Thr98 allele was more common in female patients than in female controls (odds ratio 1.69, 95% CI 1.19–2.40, *P *= 0.003). Haplotype-based analyses also provided evidence for a significant association in females.

**Conclusion:**

We failed to replicate the previously reported association of Pro4Thr of the VMAT1 gene with schizophrenia. However, we obtained evidence for a possible role of the Thr98Ser in giving susceptibility to schizophrenia in women.

## Background

Vesicular monoamine transporters (VMATs) mediate accumulation of monoamines such as serotonin, dopamine, adrenaline, noradrenaline, and histamine from the cytoplasm into storage organelles with an absolute dependence on a vacuolar ATPase-generated proton gradient to transport the cationic amine substrates into the storage organelle in exchange for protons [1-3]. There are two isoforms of VMATs identified in rats and humans [4-8]: VMAT1 (previously known as chromaffin granule amine transporter; CGAT) and VMAT2 (alternatively designated as synaptic vesicle monoamine transporter; SVMT). They are also the first and second members of the solute carrier family 18 (SLC18A1 and SLC18A2, respectively). These proteins share 60% sequence identity; however, they demonstrate a range of differences in their physiologic and pharmacologic properties. VMAT1 is expressed primarily in neuroendocrine cells such as the adrenal medulla and pineal gland, while VMAT2 is expressed in all aminergic neurons in the mammalian CNS [6,9,10]. The expression of the two isoforms in a given cell type is usually, but not always, mutually exclusive [2,11]. Furthermore, the two isoforms differ in recognition of substrates (e.g., histamine) and sensitivity to inhibitors such as tetrabenazine and methamphetamine [12]. Since biogenic amines play critical roles in consciousness, mood, thought, motivation, cognition, perception, and autonomic responses, alterations in genes encoding VMATs might play an important role in the pathogenesis of neuropsychiatric diseases including schizophrenia.

With respect to the human VMAT2 gene, we previously reported exon/intron boundaries, novel polymorphisms, and association analysis with schizophrenia; however, we did not find any polymorphism that resulted in an amino acid change [13]. In addition, we failed to obtain evidence for a significant association of the detected polymorphisms with schizophrenia [13]. The other VMAT, VMAT1, is also an attractive candidate gene for schizophrenia not only because it plays a critical role in the maintenance of monoaminergic endocrine systems but also it maps to chromosome 8p21.3 [14], a locus with strong evidence for linkage with schizophrenia [15-21]. In accordance with the possible role of the VMAT1 gene in schizophrenia, a recent study reported that an SNP in exon 3 of the gene that results in an amino acid change (277C > A resulting in Pro4Thr) was significantly associated with schizophrenia [22]. The C/C genotype (homozygosity for proline residue at codon 4) occurred in 21.4% of the schizophrenic group and only 2.6% of the control group. The A/A genotype (homozygosity for threonine), on the other hand, occurred in 28.6% of the schizophrenic group and 73.6% of the control group. Such a dramatic difference in one polymorphism of the VMAT1 gene in a Caucasian population prompted us to attempt replication of this finding in a Japanese population. In addition, we examined other non-synonymous polymorphisms in the VMAT1 gene for association with schizophrenia.

## Methods

### Subjects

Subjects were 354 patients with schizophrenia (212 males, mean age of 44.0 years [SD 13.7]) and 365 healthy controls (113 males, mean age of 39.7 years [SD 14.1]). All subjects were biologically unrelated Japanese and recruited from the same geographical area (Western part of Tokyo Metropolitan). Consensus diagnosis by at least two psychiatrists was made for each patient according to the Diagnostic and Statistical Manual of Mental Disorders, 4th edition (DSM-IV) criteria [23] on the basis of unstructured interviews and information from medical records. The majority of the patients (318 patients, 90%) had a history of admission to a psychiatric hospital. Mean age of onset was 24.4 years [SD 8.6]. Twenty-nine percent of the patients (102 patients) had a family history of schizophrenia spectrum disorders within the second degree relatives. The controls were healthy volunteers recruited from hospital staffs and their associates. Control individuals were interviewed and those who had current or past history of psychiatric treatment were not enrolled in the study. The study protocol was approved by the ethics committee at the National Center of Neurology and Psychiatry, Japan. After description of the study, written informed consent was obtained from every subject.

### Genotyping

Since genetic variations that result in an amino acid change are most likely to alter function, we searched for non-synonymous polymorphisms of the VMAT1 gene *in silico *based on the NCBI dbSNP database and found 4 well-validated SNPs with a heterozygosity value of > 0.10. They were rs2270641 (SNP1, 277C > A, Pro4Thr), rs2270637 (SNP2, 560C > G, Thr98Ser), rs1390938 (SNP3, 674C > T, Thr136Ile), and rs17092104 (SNP4, 1441G > C, Val392Leu). The numbers of base and amino acid positions were according to NM_003053 and NP_003044, respectively. Venous blood was drawn from the subjects and genomic DNA was extracted from whole blood according to the standard procedures. The SNPs were genotyped using the TaqMan 5'-exonuclease allelic discrimination assay; the assay ID (Applied Biosystems) for each SNP was C_22271506_10 for SNP1, C_2716008_1 for SNP2, C_8804621_1 for SNP3, and C_2715953_10 for SNP4. Thermal cycling conditions for polymerase chain reaction (PCR) were 1 cycle at 95°C for 10 minutes followed by 50 cycles of 92°C for 15 seconds and 60°C for 1 minute. Genotype data were read blind to the case-control status. Ambiguous genotype data were not included in the analysis.

### Statistical analysis

Deviations of genotype distributions from the Hardy-Weinberg equilibrium were assessed with the χ^2 ^test for goodness of fit. Genotype and allele distributions were compared between patients and controls by using the χ^2 ^test for independence. These tests were performed with the SPSS software ver 11 (SPSS Japan, Tokyo, Japan). Haplotype-based association analyses were examined with the COCAPHASE software ver 2.4 [24]. The expectation-maximization (EM) and "droprare" options were used. Haplotypes with frequencies less than 3 % were considered to be rare. We examined associations by permutation procedure (10,000 replications) to determine the empirical significance.

## Results

Genotype and allele distributions of the examined SNPs in patients and controls are shown in Table 1. The genotype distributions did not significantly deviate from the Hardy-Weinberg equilibrium in patients and controls for any SNPs. With respect to SNP1, there was no significant difference in genotype or allele distributions between patients and controls. Both genotype and allele distributions were approximately the same in the two groups; therefore, we failed to replicate the finding of Bly [22]. For the remaining SNPs, however, we found a significant difference in genotype and allele distributions of SNP2, but not SNP3 or SNP4, between patients and controls. For SNP2, the Thr98 (560C) allele was significantly more common in patients than in controls (*P *= 0.01, odds ratio = 1.39, 95% CI 1.09–1.77). When men and women were examined separately, genotype and allele distributions of SNP2 significantly differ in females, but not in males, between the two groups (Table 2). The excess of the Thr98 allele in patients was highly significant in females (χ^2 ^= 8.54, df = 1, *P *= 0.003, odds ratio = 1.69, 95% CI 1.19–2.40), whereas genotype and allele distributions were quite similar in male patients and controls.

**Table 1:** Genotype and allelic distributions of the VMAT1 SNPs in patients with schizophrenia and controls

dbSNP ID	Position^a^	Inter-SNP distance (bp)	Group	N	Genotype frequency (GF)			Allele frequency (AF)		Odds ratio (95% CI)	Chi-square test		
											HWE (df = 1)	GF (df = 2)	AF (df = 1)
	7883394				C/C	A/C	A/A	C	A				
SNP1 rs2270641	Exon 2	____	Patients	351	45 (0.13)	153 (0.44)	153 (0.44)	243 (0.35)	459 (0.65)	0.83 – 1.29	χ^2 ^= 0.48, *P *= 0.49	*P *= 0.95	*P *= 0.75
	Pro4Thr		Controls	360	49 (0.14)	157 (0.44)	154 (0.43)	255 (0.35)	465 (0.65)	1.04	χ^2 ^= 0.78, *P *= 0.37	χ^2 ^= 0.11	χ^2 ^= 0.10
	7881755				C/C	G/C	G/G	C	G				
SNP2 rs2270637	Exon 3	1639	Patients	352	11 (0.03)	130 (0.37)	211 (0.60)	152 (0.22)	552 (0.78)	1.09 – 1.77	χ^2 ^= 2.9, *P *= 0.09	*P *= 0.01	*P *= 0.01
	Thr98Ser		Controls	362	28 (0.08)	144 (0.40)	190 (0.52)	200 (0.28)	524 (0.72)	1.39	χ^2 ^= 0.0, *P *= 0.92	χ^2 ^= 9.09	χ^2 ^= 7.00
	7881641				C/C	T/C	T/T	C	T				
SNP3 rs1390938	Exon 3	114	Patients	352	188 (0.53)	135 (0.38)	29 (0.08)	511 (0.73)	193 (0.27)	0.70 – 1.13	χ^2 ^= 0.46, *P *= 0.50	*P *= 0.44	*P *= 0.33
	Thr136Ile		Controls	360	200 (0.56)	139 (0.39)	21 (0.06)	539 (0.75)	181 (0.25)	0.89	χ^2 ^= 0.24, *P *= 0.62	χ^2 ^= 1.62	χ^2 ^= 0.95
	7850482				G/G	G/C	C/C	G	C				
SNP4 rs17092104	Exon 13	31159	Patients	352	0 (0.00)	23 (0.07)	329 (0.93)	23 (0.03)	681 (0.97)	0.38 – 1.34	χ^2 ^= 0.40, *P *= 0.53	*P *= 0.28	*P *= 0.29
	Val392Leu		Controls	363	0 (0.00)	17 (0.05)	346 (0.95)	17 (0.02)	709 (0.98)	0.71	χ^2 ^= 0.21, *P *= 0.65	χ^2 ^= 1.16	χ^2 ^= 1.13

^a^Chromosome position was according to the dbSNP database.

HWE: Hardy-Weinberg equilibrium

P values of < 0.05 are underlined.

**Table 2:** Genotype and allele distributions of SNP2 (Thr98Ser) in patients with schizophrenia and controls for each sex

		N	Genotype distribution (frequency)								Allele distribution (frequency)						HWE	
			
*Total*			CC		GC		GG		χ^2^	*P*	C		G		χ^2^	P	χ^2^	P
	Patients	352	11	(0.03)	130	(0.37)	211	(0.60)	9.09	0.011	152	(0.22)	552	(0.78)	7.00	0.008	2.90	0.089
	Controls	362	28	(0.08)	144	(0.40)	190	(0.52)			200	(0.28)	524	(0.72)			0.01	0.921
*Male*																		
	Patients	211	9	(0.04)	79	(0.37)	123	(0.58)	2.27	0.322	97	(0.23)	325	(0.77)	0.20	0.655	0.70	0.404
	Controls	112	9	(0.08)	37	(0.33)	66	(0.59)			55	(0.25)	169	(0.75)			1.31	0.252
*Female*																		
	Patients	141	2	(0.01)	51	(0.36)	88	(0.62)	10.12	0.006	55	(0.20)	227	(0.80)	8.54	0.003	3.26	0.071
	Controls	250	19	(0.08)	107	(0.43)	124	(0.50)			145	(0.29)	355	(0.71)			0.39	0.534

HWE: Hardy-Weinberg equilibrium

Significant P values are underlined.

Pair-wise linkage disequilibrium values between neighbouring SNPs are shown in Table 3. Fairly tight linkage disequilibrium was observed in any pair of the SNPs. We obtained no significant difference in haplotype frequencies for two-, three-, or four-marker analyses between patients and controls in males (data not shown). In females, however, we obtained significant differences in estimated haplotype distributions for any comparisons when SNP2 was included in the analysis (Table 4). The most significant result was obtained by the two-marker haplotype (C-C) consisting of SNP2 and SNP3 (permutation *P *= 0.007).

**Table 3:** Pair-wise linkage disequilibrium between neighbouring SNPs in the VMAT1 gene

	SNP1	SNP2	SNP3	SNP4
	rs2270641	rs2270637	rs1390938	rs17092104
SNP1		0.70	0.99	1.00
SNP2	0.29		1.00	1.00
SNP3	0.19	0.12		1.00
SNP4	0.02	0.01	0.01	

Upper diagonal figures are D' and lower diagonal figures are *r*^2^.

Pairs in LD (D' > 0.8 or *r*^2 ^> 0.8) are underlined.

**Table 4:** Estimated haplotype frequencies and significance of differences between patients and controls in females

Haplotype				Haplotype frequency (%)		*P*-values		
SNP1	SNP2	SNP3	SNP4	Patients	Controls	Individual	Global	Permutation global
C	G	/	/	0.21	0.14	0.015	0.004	0.008
C	C	/	/	0.13	0.23	0.002		
/	C	C	/	0.20	0.29	0.003	0.012	0.007
C	G	C	/	0.21	0.14	0.017	0.010	0.011
C	C	C	/	0.14	0.23	0.002		
/	C	C	C	0.20	0.29	0.004	0.021	0.012
C	G	C	C	0.21	0.14	0.025	0.012	0.021
C	C	C	C	0.14	0.23	0.003		

Haplotype individual p-values of < 0.05 are listed.

## Discussion

We failed to replicate the finding of Bly [22] who reported a significant association between the Pro4Thr polymorphism (SNP1) of the VMAT1 gene and schizophrenia. This discrepancy may be attributable to ethnic differences in the effects of SNP1 between Caucasians and Asians. The possibility of a type-II error is unlikely because our sample size had a power of approximately 100% to detect the difference in the frequency of C/C genotype reported in Bly's study (21.4% in patients and 2.6% in controls). Moreover, both the genotype and allele distributions of SNP1 were almost the same in our patients and controls. An alternative possibility might be that the finding of Bly [22] had arisen by chance due to the small sample size (28 schizophrenics and 38 controls) and thus obtained evidence of statistical significance was not strong (*P *= 0.036) in spite of the marked difference in the frequency of C/C genotype between patients and controls in his sample.

When additional SNPs were genotyped, we found that the 98Thr (560C) allele of SNP2 was significantly increased in schizophrenics compared to controls, although no significant results were obtained for SNP3 or SNP4. This significant excess of the 98Thr allele in patients was observed in females, but not in males, suggesting that the Thr98 allele has a sexually dimorphic effect of giving susceptibility to schizophrenia. Considering that the frequency of the 98Thr allele was greater than the 98Ser allele, it might be more appropriate to infer that the 98Ser allele has a protective effect against the development of schizophrenia. Haplotype-based analyses also yielded several significant differences in haplotype frequencies between female patients and controls only when SNP2 was included in the analysis, providing further support for the possible role of SNP2 in female schizophrenia. However, since we examined only non-synonymous SNPs that had been deposited in the public database (dbSNP) and did not perform polymorphism screening, we may have missed unknown functional polymorphisms. It is possible that such unknown polymorphisms nearby which are in linkage disequilibrium to the SNP2 might be "truly" responsible in giving susceptibility to schizophrenia.

The Thr98Ser polymorphism may affect the processing and overall function of VMAT1 through altering cell signaling and protein trafficking pathways. The human VMAT1 gene is composed of 18 exons which encode 525 amino acids [5]. There are 12 predicted transmembrane domains in the VMAT1 secondary structure and a large luminal loop between transmembrane domains 1 and 2. The Thr98Ser polymorphism is located on this luminal loop, in which there are three potential sites for N-linked glycosylation (asparagines residues at codons 58, 87 and 104) [6]. This loop is the main site of N-glycosylation on the VMAT1 protein, which is believed to regulate targeting of the protein. Since the Thr98Ser is closely located to N-linked glycosylation sites, it is possible that the Thr98Ser polymorphism may affect glycosylation status. Another possibility is that the Thr98Ser polymorphism may lead to altered phosphorylation in the VMAT1 protein, since serine and threonine residues play a central role in phosphorylation (activation/inactivation) of proteins. Indeed, some serine residues have been shown to undergo phosphorylation in the isoform protein VMAT2 [25]. However, conclusions remain purely speculative and additional research on protein structure, cell signaling, and protein trafficking pathways within VMAT1 are required.

We detected a significant association between the VMAT1 gene and schizophrenia only in females. This observation is not surprising, because there is substantial evidence for sex differences in the pathogenesis and pathophysiology of schizophrenia, which may have arisen from interplay between sex hormones and other developmental factors [26]. Indeed, there are several other genes (e.g., ZDHHC8 [27] and chimerin 2 [28]) that have been suggested to have a sexually dimorphic effect on the development of schizophrenia. Furthermore, there is evidence for crucial regulation by ovarian steroids on the expression of the VMAT2 gene [29]. Although there is little information on such regulation for the VMAT1 gene, it is possible that similar regulation exists, which may be related to our observation of the differential effect of the VMAT1 gene between males and females.

Recently, Lohoff et al [30] reported a significant association between the VMAT1 gene and bipolar I disorder. They genotyped three non-synonymous SNPs (Thr4Pro, Thr98Ser, and Thr136Ile) and 4 non-coding SNPs, and found that allele frequencies in the Thr136Ile, and polymorphisms in the promoter region and intron 8 differed significantly between patients and controls of European descent. Although the associated SNP was again different with our results, the results of Lohoff et al [30] and ours might support the view that schizophrenia and bipolar has several similarities and share susceptibility genes [31].

## Conclusion

In conclusion, although we failed to replicate the finding of Bly [22], our results suggest that another amino acid substitution (Thr98Ser) of the VMAT1 gene may have a sexually dimorphic effect of giving susceptibility to schizophrenia in the Japanese population. If our results are replicated, further investigations on VMAT1 function may elucidate molecular mechanisms of schizophrenia, permitting the development of novel therapeutic agents.

## Competing interests

The author(s) declare that they have no competing interests.

## Authors' contributions

MR, YI, HitK, and TS performed genotyping and statistical analyses. MR helped to draft the manuscript. HH, KA, and OS recruited and assessed the subjects and helped to draft the manuscript. HirK designed the study, recruited the subjects, and drafted the manuscript. All authors read and approved the final manuscript.
